# A Comprehensive Review of Nanoparticle-Based Drug Delivery for Modulating PI3K/AKT/mTOR-Mediated Autophagy in Cancer

**DOI:** 10.3390/ijms26051868

**Published:** 2025-02-21

**Authors:** Md Ataur Rahman, Maroua Jalouli, Sujay Kumar Bhajan, Mohammed Al-Zharani, Abdel Halim Harrath

**Affiliations:** 1 Department of Oncology, Karmanos Cancer Institute, Wayne State University, Detroit, MI 48201, USA; 2 Department of Biology, College of Science, Imam Mohammad Ibn Saud Islamic University (IMSIU), Riyadh 11623, Saudi Arabia; mejalouli@imamu.edu.sa (M.J.); mmylzahrani@imamu.edu.sa (M.A.-Z.); 3 Department of Biotechnology and Genetic Engineering, Faculty of Life Sciences, Bangabandhu Sheikh Mujibur Rahman Science and Technology University, Gopalganj 8100, Bangladesh; sujaybge@gmail.com; 4 Zoology Department, College of Science, King Saud University, Riyadh 11451, Saudi Arabia; hharrath@ksu.edu.sa

**Keywords:** nanoparticles, drug delivery, PI3K/AKT/mTOR, autophagy, cancer therapy, targeted therapy

## Abstract

The phosphoinositide 3-kinase (PI3K)/AKT/mammalian target of the rapamycin (mTOR) pathway plays a crucial role in the regulation of autophagy, a cellular mechanism vital for homeostasis through the degradation of damaged organelles and proteins. The dysregulation of this pathway is significantly associated with cancer progression, metastasis, and resistance to therapy. Targeting the PI3K/AKT/mTOR signaling pathway presents a promising strategy for cancer treatment; however, traditional therapeutics frequently encounter issues related to nonspecific distribution and systemic toxicity. Nanoparticle-based drug delivery systems represent a significant advancement in addressing these limitations. Nanoparticles enhance the bioavailability, stability, and targeted delivery of therapeutic agents, facilitating the precise modulation of autophagy in cancer cells. Functionalized nanoparticles, such as liposomes, polymeric nanoparticles, and metal-based nanocarriers, facilitate targeted drug delivery to tumor tissues, minimizing off-target effects and improving therapeutic efficacy. These systems can deliver multiple agents concurrently, enhancing the modulation of PI3K/AKT/mTOR-mediated autophagy and related oncogenic pathways. This review examines advancements in nanoparticle-mediated drug delivery that target the PI3K/AKT/mTOR pathway, emphasizing their contribution to improving precision and minimizing side effects in cancer therapy. The integration of nanotechnology with molecularly targeted therapies presents substantial potential for addressing drug resistance. Future initiatives must prioritize the optimization of these systems to enhance clinical translation and patient outcomes.

## 1. Introduction

Autophagy is a crucial mechanism that conserves cellular integrity by degrading and recycling damaged organelles and proteins, thereby maintaining cellular homeostasis [1,2]. Autophagy functions in a dual capacity in cancer, serving as a tumor suppressor in early stages and as a tumor promoter in advanced stages, where it supplies cancer cells with essential metabolic resources for survival under stress [3]. The phosphoinositide 3-kinase (PI3K)/AKT/mammalian target of rapamycin (mTOR) signaling pathway plays a central role in the regulation of autophagy, affecting cellular growth, survival, and metabolic processes [4]. Dysregulation of this pathway is commonly observed in cancer, contributing to tumorigenesis, metastasis, and resistance to standard therapies. However, cancer remains a major global health challenge, with its progression, metastasis, and therapy resistance frequently associated with complex molecular mechanisms that regulate cellular behavior.

**Figure 1:**
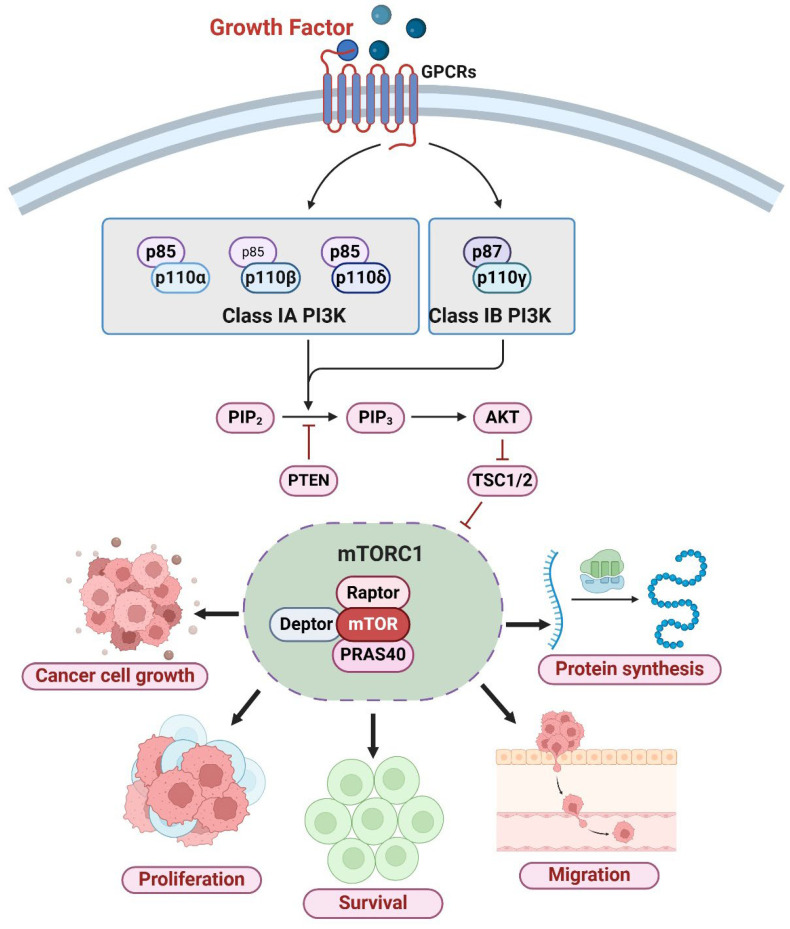
The PI3K/AKT/mTOR pathway in Cancer. After a growth factor binds to its receptor, the route activates Class I phosphoinositide 3-kinases. Receptor tyrosine kinases activate Class IA PI3K, while G-protein-coupled receptors activate Class IB. Both classes convert PIP2 into PIP3, which recruits and activates plasma membrane AKT. Activated AKT phosphorylates and suppresses TSC1/2, a negative regulator of mTORC1. This blockage activates mTORC1, a protein synthesis, cell metabolism, and growth master regulator. The mTORC1 complex boosts translation and cancer growth, proliferation, and survival. The mechanism also aids cancer migration and metastasis. The figure was created using the BioRender.com online commercial platform.

The PI3K/AKT/mTOR signaling pathway is a pivotal regulator of cellular growth, survival, metabolism, and autophagy [5]. This pathway is initiated by extracellular signals, such as growth factors, cytokines, and nutrients, which activate intracellular cascades essential for cellular homeostasis. The PI3K/AKT/mTOR system exerts a negative regulation on autophagy, primarily via mTORC1, which suppresses the ULK1/2 (unc-51-like kinase 1/2) complex, a crucial starter of autophagy [6]. In the presence of ample nutrition and growth stimuli, PI3K phosphorylates PIP2 (phosphatidylinositol-4,5-bisphosphate) to produce PIP3 (phosphatidylinositol-3,4,5-trisphosphate), thereby activating AKT [7]. AKT subsequently phosphorylates and inhibits TSC1/TSC2 (tuberous sclerosis complex 1/2), resulting in mTORC1 activation and autophagy inhibition [8]. Conversely, during stress or hunger, PI3K/AKT signaling is diminished, resulting in mTORC1 inhibition, which triggers autophagy to reutilize intracellular components for energy generation [9]. Dysregulation of the PI3K/AKT/mTOR pathway is frequently noted in cancer, resulting in unregulated cell proliferation, survival, and resistance to therapy (Figure 1) [10]. Hyperactivation of mTORC1 inhibits autophagy, facilitating tumor growth by obstructing the breakdown of oncogenic proteins [11]. Nonetheless, autophagy may also facilitate the survival of cancer cells under stressful conditions, including chemotherapy or hypoxia [12]. Utilizing small molecule inhibitors (e.g., PI3K, AKT, and mTOR inhibitors) to target this system has emerged as a viable method for cancer therapy [13]. The simultaneous regulation of PI3K/AKT/mTOR signaling and autophagy is under investigation to address resistance mechanisms and enhance therapeutic efficacy in cancer treatment [14]. Therefore, comprehending the regulatory interaction between PI3K/AKT/mTOR signaling, autophagy, and cancer is essential for formulating innovative therapeutic strategies.

The PI3K/AKT/mTOR signaling system serves as a principal negative regulator of autophagy, predominantly via mTORC1 [5]. In nutrient-rich situations, PI3K phosphorylates PIP2 to become PIP3, thereby activating AKT, which then phosphorylates and inhibits TSC1/TSC2 [15]. This inhibition results in mTORC1 activation, which inhibits autophagy by phosphorylating ULK1, hence obstructing autophagosome formation [16]. In contrast, under stress circumstances, such as food restriction or hypoxia, PI3K/AKT signaling is suppressed, resulting in mTORC1 inhibition, which activates ULK1 and initiates autophagy [17]. This transition allows cells to decompose and reutilize intracellular constituents to sustain energy equilibrium and viability. In cancer, the hyperactivation of the PI3K/AKT/mTOR pathway inhibits autophagy, facilitating tumor formation; conversely, in certain instances, the activity of autophagy aids cancer cells in enduring therapy-induced stress [18]. Consequently, targeting this route offers a prospective therapeutic approach for regulating autophagy in cancer therapy.

Nanotechnology provides a significant solution to these challenges via the creation of nanoparticle-based drug delivery systems [19]. Nanoparticles exhibit distinctive physicochemical characteristics, including their nanoscale dimensions, high surface area-to-volume ratio, and potential for functionalization with targeting ligands [20]. These attributes allow nanoparticles to transport therapeutic agents to cancer cells with high specificity and efficiency, thereby reducing off-target effects. Functionalized nanoparticles, such as liposomes, polymeric nanoparticles, and metal-based nanocarriers, can be designed to improve drug stability, bioavailability, and controlled release [21].

**Figure 2:**
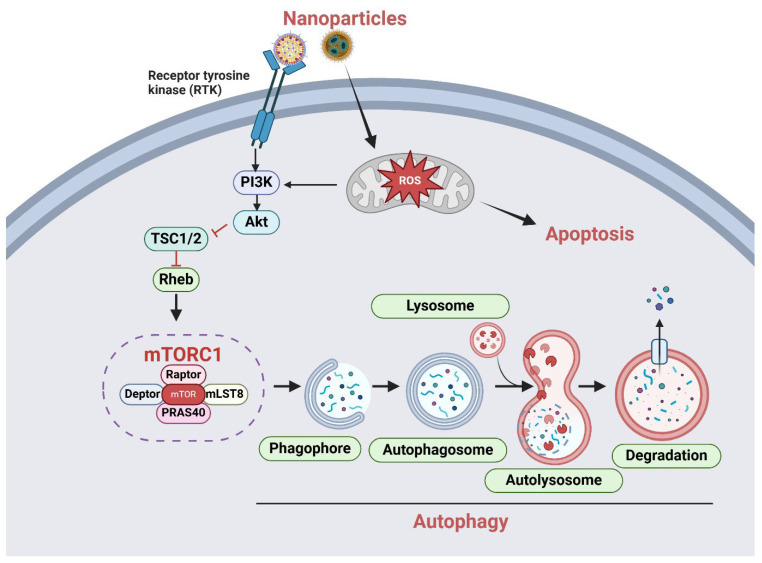
PI3K/AKT/mTOR and autophagy-mediated nanoparticle signaling in cancer. Nanoparticles bind with receptor tyrosine kinases (RTKs) on cancer cells, initiating the activation of the PI3K/AKT/mTOR signaling cascade. Activated PI3K produces PIP3, which attracts and activates AKT. AKT phosphorylates and inhibits the TSC1/2 (tuberous sclerosis complex), hence obstructing the inhibition of Rheb, a small GTPase that stimulates mTORC1 and autophagy-mediated cell death. Nanoparticles play a significant role in cancer therapy by modulating reactive oxygen species (ROS) levels and inducing apoptosis in cancer cells. The interactions among nanoparticles, ROS production, and apoptosis can occur either independently or simultaneously in cancer cells, depending upon cellular context, NP characteristics, and microenvironmental factors. The figure was created using the BioRender.com online commercial platform.

Nanoparticle-based systems exhibit significant potential for targeted delivery to tumor tissues, primarily due to the enhanced permeability and retention (EPR) effect [22]. The EPR effect plays a crucial role in nanoparticle-based drug delivery for cancer treatment. Due to the leaky vasculature and poor lymphatic drainage in tumor tissues, nanoparticles accumulate preferentially in the tumor microenvironment [23]. This selective accumulation enhances the therapeutic efficacy of anticancer agents while reducing off-target effects and systemic toxicity, which are major concerns in conventional chemotherapy. Additionally, nanoparticles can be engineered to react to stimuli, including pH, temperature, or redox conditions, facilitating targeted drug release [24]. This feature is beneficial for modulating autophagy by allowing targeted intervention in the PI3K/AKT/mTOR pathway within the tumor microenvironment. The interaction of nanoparticle signaling with the PI3K/AKT/mTOR and autophagy pathways highlights the dual function of autophagy in cancer, serving as both a pro-survival mechanism and a facilitator of apoptosis (Figure 2). Recognizing this dynamic interplay is essential for enhancing nanoparticle-based treatment approaches that target cancer growth and progression.

Recent advancements have occurred in the design and application of nanoparticle-based drug delivery systems targeting the PI3K/AKT/mTOR pathway [25]. Preclinical studies indicate that these systems may improve the effectiveness of cancer therapies and reduce adverse effects. Liposomal formulations of PI3K inhibitors demonstrate enhanced bioavailability and tumor accumulation relative to free drugs [26]. Polymeric nanoparticles containing dual inhibitors of PI3K and mTOR have shown promising outcomes in preclinical cancer models [27]. Metal-based nanocarriers, including gold and iron oxide nanoparticles, have been investigated for their potential in delivering therapeutic agents and enhancing imaging-guided therapy [28]. The advancements underscore the versatility and potential of nanoparticle-based platforms in cancer treatment. The combination of nanotechnology and molecularly targeted therapy signifies a significant advancement in cancer treatment [29]. Nanoparticle-based systems present a promising strategy to enhance the effectiveness of cancer therapies by selectively influencing the PI3K/AKT/mTOR pathway and other tumor-promoting processes, thereby overcoming the limitations of conventional treatments [30]. This method may improve the accuracy, effectiveness, and safety of cancer treatments, thereby enhancing patient outcomes. Ongoing research will facilitate the clinical translation of these systems, leading to more effective and personalized cancer treatments.

Thus, nanoparticle-mediated drug delivery systems offer a viable approach for targeting the PI3K/AKT/mTOR signaling pathway in cancer treatment. These systems have the potential to transform cancer treatment by addressing the limitations of conventional therapies, enhancing precision, minimizing side effects, and overcoming drug resistance. This review presents an overview of advancements in nanoparticle-based strategies for modulating PI3K/AKT/mTOR-mediated autophagy in cancer. This investigation aims to contribute to ongoing efforts in utilizing nanotechnology to enhance cancer treatment outcomes by emphasizing recent advancements and future directions.

## 2. Nanoparticles-Based Drug Delivery in Cancer Management

Nanoparticles (NPs) represent a significant advancement in cancer management, providing novel approaches to address the limitations of traditional cancer treatments. Nanostructures, generally measuring between 1 and 100 nanometers, exhibit distinctive physicochemical properties, including a high surface area-to-volume ratio, adjustable size, and potential for functionalization [31]. These features facilitate efficient drug loading, controlled release, and targeted delivery of therapeutic agents to cancer cells, thereby enhancing therapeutic outcomes and reducing systemic side effects.

### 2.1. Classification and Categories of Nanoparticles

**Figure 3:**
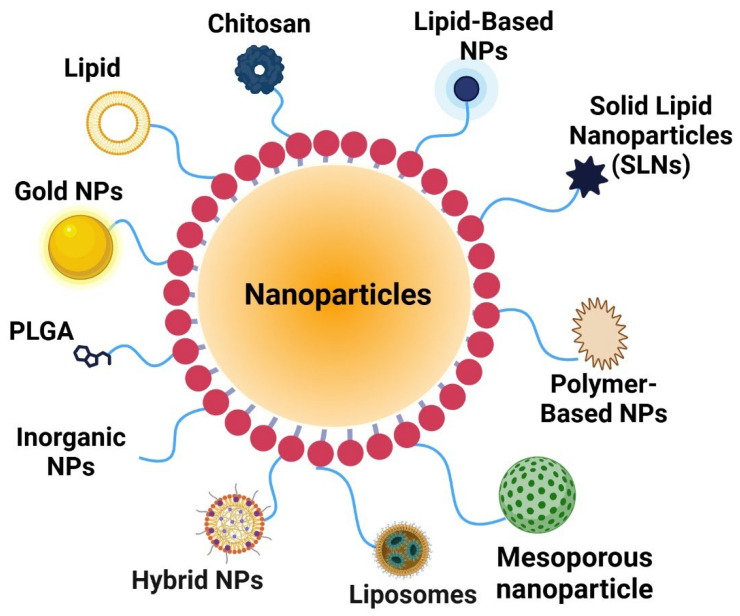
Key nanoparticle categories for cancer drug delivery. Many nanoparticles are used in cancer therapy and a therapeutic use. Liposomes and solid lipid nanoparticles (SLNs) are intensively explored for encapsulating hydrophilic and hydrophobic medicines. Another important group is polymer-based nanoparticles, such as PLGA, chitosan, and dendrimers. PLGA nanoparticles are biocompatible and biodegradable, making them excellent for prolonged drug release. Chitosan, a natural polymer, is mucoadhesive and ideal for targeted therapy. Dendrimers’ branching architectures enable precise drug loading and functionalization, improving targeting. Gold nanoparticles (AuNPs), silica nanoparticles, and quantum dots are recognized for their unique functions. In photothermal therapy, gold nanoparticles absorb light and generate heat to kill cancer cells. Silica nanoparticles’ porous architecture allows high drug loading, while quantum dots’ fluorescence allows imaging-guided drug delivery. Hybrid nanoparticles combine organic and inorganic benefits. Lipid-coated gold nanoparticles combine biocompatibility with photothermal characteristics, improving cancer treatment. The figure was created using the BioRender online commercial platform.

Nanoparticles are categorized into various types according to their composition and structure (Figure 3). Lipid-based nanoparticles, such as liposomes and solid lipid nanoparticles (SLNs), represent some of the most thoroughly researched carriers in cancer therapy. Liposomes are composed of phospholipid bilayers that encapsulate hydrophilic and hydrophobic drugs, facilitating stable delivery. Solid lipid nanoparticles (SLNs) provide improved stability and facilitate controlled drug release. Polymer-based nanoparticles, such as poly(lactic-co-glycolic acid) (PLGA), chitosan, and dendrimers, are extensively employed in drug delivery systems. PLGA nanoparticles exhibit biocompatibility and biodegradability, rendering them suitable for sustained drug release applications. Chitosan, obtained from natural sources, exhibits mucoadhesive properties and is frequently utilized in targeted cancer therapies. Dendrimers possess branched architectures that facilitate accurate drug loading and functionalization aimed at targeted cancer therapy. Inorganic nanoparticles, including gold, silica, and quantum dots, are widely studied for their distinct optical and electronic characteristics. Gold nanoparticles (AuNPs) are employed in photothermal therapy, a process in which they absorb light and transform it into heat to induce cytotoxicity in cancer cells. Silica nanoparticles offer a porous architecture conducive to high drug loading, whereas quantum dots facilitate imaging-guided drug delivery owing to their fluorescence characteristics. Hybrid nanoparticles integrate organic and inorganic materials to utilize the benefits of each component. Lipid-coated nanoparticles combine the biocompatibility of lipids with the photothermal properties of gold, thereby improving their therapeutic potential in cancer treatment.

### 2.2. Benefits of Drug Delivery Utilizing Nanoparticles

Nanoparticle-based drug delivery systems offer numerous advantages compared to conventional chemotherapy and radiotherapy, effectively addressing various limitations inherent in traditional cancer treatments. The enhanced permeability and retention (EPR) effect facilitates the preferential accumulation of nanoparticles in tumor tissues, attributable to their leaky vasculature and inadequate lymphatic drainage. This passive targeting mechanism increases the accumulation of therapeutic agents at the tumor site, minimizing exposure to healthy tissues. Surface modification of nanoparticles through functionalization with ligands, including antibodies, peptides, or aptamers, facilitates the active targeting of specific cancer cell receptors. This method enhances therapeutic effectiveness while minimizing off-target effects. Nanoparticles enhance the pharmacokinetics of encapsulated drugs by minimizing systemic toxicity and preventing premature degradation, thereby facilitating controlled release profiles that ensure sustained therapeutic effects.

### 2.3. Application of Nanoparticles in Cancer Treatment

Nanoparticles exhibit significant potential for the delivery of chemotherapeutic agents, gene therapies, and immunotherapies. Liposomal formulations, exemplified by Doxil (liposomal doxorubicin), have received FDA approval for the treatment of cancer. Polymer-based and inorganic nanoparticles are being extensively studied for the delivery of siRNA, CRISPR-Cas9, and checkpoint inhibitors, presenting new opportunities for cancer immunotherapy. Thus, nanoparticle-based drug delivery systems signify a significant advancement in cancer management, overcoming the limitations of traditional therapies and facilitating more precise, effective, and patient-centered treatments. Their adaptability and adjustable characteristics guarantee their essential function in the future of oncology.

## 3. Targeting PI3K/AKT/mTOR-Mediated Autophagy with Nanoparticles

Nanoparticles have emerged as an effective tool for the precise control of the PI3K/AKT/mTOR pathway, significantly contributing to cancer therapy. Nanoscale carriers can be designed to deliver small molecule inhibitors, siRNA, or gene-editing tools with great specificity, thereby improving therapeutic efficacy and reducing off-target effects.

### 3.1. Small Molecule Inhibitors

Nanoparticles have gained significant attention in cancer therapy due to their ability to selectively deliver therapeutic agents, improving drug bioavailability and treatment outcomes. The PI3K/AKT/mTOR pathway is a key regulator of cancer progression, influencing various cellular processes, including autophagy [32]. Utilizing nanoparticles to target this pathway has become a promising approach for enhancing cancer treatment efficacy. Table 1 presented illustrates several nanoparticles that specifically target the PI3K/AKT/mTOR-mediated autophagy pathway across various cancer types. Liposomes containing PI3K inhibitors, such as LY294002, represent an extensively researched category of nanoparticle systems [33]. Liposomal formulations inhibit PI3K signaling, leading to the suppression of autophagy and the promotion of apoptosis in cancers, including breast and prostate cancer. Polymeric nanoparticles containing rapamycin, a recognized mTOR inhibitor, are employed to target glioblastoma, as mTORC1 inhibition decreases autophagic flux and impedes tumor survival [34]. Gold nanoparticles containing AKT inhibitors, including AKT Inhibitor VIII, selectively inhibit AKT phosphorylation in ovarian cancer, enhancing chemosensitivity and decreasing autophagy-mediated resistance [35]. Silica nanoparticles loaded with LY294002 improve the delivery of PI3K inhibitors to lung cancer cells, thereby enhancing chemotherapeutic efficacy through the inhibition of autophagic processes [36]. Everolimus, an mTOR inhibitor, has been effectively utilized in solid lipid nanoparticles (SLNs) for targeted pancreatic cancer therapy. By incorporating Everolimus into SLNs, its solubility and bioavailability are enhanced, leading to improved therapeutic efficacy. Additionally, Everolimus suppresses autophagy, a cellular survival mechanism that cancer cells often exploit for growth under stress conditions. By inhibiting autophagy, Everolimus contributes to tumor suppression, making it a promising strategy in pancreatic cancer treatment [37]. Dendrimers containing Wortmannin, a PI3K inhibitor, enhance anti-tumor activity and apoptosis in breast cancer cells through the disruption of autophagy [38]. Rapamycin is encapsulated in iron oxide nanoparticles to inhibit mTOR activity in hepatocellular carcinoma, thereby enhancing therapeutic response [39]. PLGA, (poly(lactic-co-glycolic acid)) nanoparticles incorporating MK2206, an AKT inhibitor, have demonstrated an increased anticancer efficacy in non-small cell lung cancer through the disruption of autophagic mechanisms [40]. Chitosan nanoparticles containing GDC-0941 inhibit PI3K in triple-negative breast cancer, thereby enhancing chemotherapy sensitivity [41]. Meanwhile, carbon nanotubes loaded with Torin 1 suppress both mTORC1 and mTORC2 in colorectal cancer, leading to decreased tumor cell viability and improved treatment outcomes [42].

### 3.2. siRNA and Gene Therapy

Nanoparticles can improve siRNA and gene therapy delivery to the PI3K/AKT/mTOR-mediated autophagy pathway in cancer treatment [43]. Liposomes, polymeric nanoparticles, gold nanoparticles, dendrimers, MSNs, polymeric micelles, SLNs, calcium phosphate nanoparticles, graphene oxide nanoparticles, and nanogels have different drug loading, stability, release profiles, and tumor-specific targeting. To improve therapeutic outcomes, toxicity, circulatory stability, and drug-loading capacity must be addressed. Table 2 highlights various types of nanoparticles, their potential for gene therapy via siRNA delivery, and their role in targeting the PI3K/AKT/mTOR-mediated autophagy pathway in cancer treatment. Bilayer membrane-forming phospholipid and cholesterol nanoparticles are liposomes. These nanoparticles can carry siRNA to cancer cells, where it silences PI3K/AKT/mTOR signaling pathway components [44]. Biocompatible liposomes have been widely researched for gene transfer because they protect siRNA and provide regulated release. Liposomes quiet PI3K/AKT/mTOR pathway components to decrease autophagy in cancer cells, potentially improving chemotherapy and radiation therapy [45]. Liposomes are rapidly cleared by the immune system, limiting their therapeutic usefulness. Polymeric nanoparticles, produced from biodegradable polymers, like PEG or PLGA, have lengthy circulation periods and may load enormous amounts of siRNA. These nanoparticles can be created for controlled release to deliver PI3K/AKT/mTOR pathway siRNA to tumor cells over time. Polymeric nanoparticles deliver siRNA targeting the PI3K/AKT/mTOR pathway to prevent autophagy in cancer cells, enhancing conventional therapies [46]. Despite their benefits, some polymer components may be hazardous; therefore, formulation processes must be performed carefully. Their optical characteristics, biocompatibility, and simplicity of surface functionalization make gold nanoparticles (AuNPs) popular drug delivery candidates. To target cancer cells, these nanoparticles can be functionalized with ligands or compounds, like siRNA. Gold nanoparticles can silence PI3K/AKT/mTOR genes, interrupting autophagy and boosting cancer cell death [47]. Gold nanoparticles stabilize siRNA during circulation, blocking ribonuclease breakdown. Gold nanoparticles can be hazardous at high doses, limiting their therapeutic use.

Nanoscale, highly branching dendrimers have well-defined structures and functionalized surfaces. These nanoparticles are perfect for PI3K/AKT/mTOR siRNA delivery due to their high loading capacity. Dendrimers efficiently encapsulate and release siRNA, silencing cancer cell genes [48]. Targeting ligands can modify the surface for tumor-specific delivery, improving treatment efficacy. Dendrimers provide benefits, but their synthesis is complicated, and they can be harmful at high dosages. Mesoporous silica nanoparticles (MSNs) are ideal for drug delivery due to their high surface area and variable pore diameters. MSNs loaded with siRNA can target tumor cells and silence PI3K/AKT/mTOR pathway components and modulate autophagy [49]. MSNs can release siRNA continuously, ensuring long-lasting effects on the tumor microenvironment [50]. However, silica-based nanoparticles may cause inflammation and tissue toxicity, which must be addressed during formulation. Amphiphilic copolymers create self-assembling particles called polymeric micelles. Encapsulating hydrophobic medicines or siRNA in their core, these nanoparticles are used for regulated drug delivery. PI3K/AKT/mTOR siRNA delivery by polymeric micelles has been investigated for gene therapy [50]. These nanoparticles improve siRNA stability and bioavailability, boosting cancer cell treatment. Polymeric micelles have minimal toxicity and good pharmacokinetics, but their blood circulation stability must be improved to maximize their therapeutic potential.

Solid lipid nanoparticles (SLNs) have a solid lipid core and a surfactant layer. Biocompatible SLNs are stable and prevent siRNA from undergoing enzymatic degradation [51]. These nanoparticles deliver siRNA targeting the PI3K/AKT/mTOR pathway, which may reverse autophagy therapy resistance. SLNs preserve siRNA but have low drug-loading capabilities, limiting their use for therapeutic siRNA delivery. Calcium and phosphate ions form calcium phosphate nanoparticles. Biodegradability and siRNA encapsulation make these nanoparticles promising medication delivery vehicles. Calcium phosphate nanoparticles can silence PI3K/AKT/mTOR pathway components in cancer cells without harming them, making them intriguing gene therapy candidates [36]. Calcium phosphate nanoparticles’ poor release rates limit siRNA delivery’s therapeutic efficacy. The two-dimensional lattice of carbon atoms in graphene is used to make graphene oxide nanoparticles. These nanoparticles have a high surface area, biocompatibility, and ability to functionalize with several biomolecules, including siRNA. Graphene oxide nanoparticles can block cancer cell autophagy with PI3K/AKT/mTOR siRNA [52]. They have great drug-loading capacity and stability, but their high-dose cytotoxicity and inclination to agglomerate in biological systems must be addressed. Hydrophilic, crosslinked polymeric nanogels can contain hydrophilic and hydrophobic substances. Highly biocompatible nanoparticles can be made to release siRNA continuously [53]. In cancer treatment, nanogels deliver siRNA targeting the PI3K/AKT/mTOR pathway to control autophagy and improve results. Nanogels can be modified for appropriate release rates, providing effective therapy [53]. Nanogels without surface modification have little cellular absorption, which limits their efficacy.

### 3.3. Combination Therapies

Nanoparticle-assisted combination therapies present a promising strategy for improving the efficacy of chemotherapeutic drugs by precisely modulating autophagy and counteracting drug resistance mechanisms. These nanoparticles modulate key pathways, such as PI3K/AKT/mTOR, to overcome chemoresistance, improve drug delivery, and enhance cytotoxic effects on various cancer types, as presented in Table 3. Liposomes are a widely utilized nanoparticle delivery technique in cancer therapy, owing to their biocompatibility and capacity to encapsulate both hydrophilic and hydrophobic pharmaceuticals. Doxorubicin-encapsulated liposomes specifically target the PI3K/AKT/mTOR pathway, regulating autophagy through the inhibition of mTOR signaling [54]. This inhibition diminishes the cancer cells’ capacity to endure stress, enhancing the lethal effects of the encapsulated doxorubicin. This nanoparticle formulation, in conjunction with doxorubicin, has demonstrated the ability to increase cell death in multiple malignancies, such as breast, lung, and ovarian cancers, by overcoming chemoresistance and facilitating apoptosis. Polymeric nanoparticles (PNPs) are designed to provide medications with enhanced stability and regulated release. These nanoparticles can selectively target the PI3K/AKT/mTOR pathway, consequently regulating autophagy and augmenting the efficacy of chemotherapeutic drugs, such as paclitaxel [35]. Inhibiting autophagy via mTOR suppression in cancer cells enhances therapeutic efficacy. In colorectal, pulmonary, and prostate malignancies, PNPs can synergistically augment the effectiveness of chemotherapy, diminishing tumor size and enhancing patient outcomes by circumventing drug resistance pathways. Gold nanoparticles (AuNPs) are extensively investigated for their superior biocompatibility, facile functionalization, and capability to selectively transport medications to tumor locations. AuNPs suppress autophagy by targeting the AKT/mTOR pathway, a vital mechanism for cancer cell survival under stress. When utilized alongside chemotherapeutic medicines, such as cisplatin, AuNPs can enhance the cytotoxicity of the drug, offering a more effective therapy strategy for malignancies including lung, ovarian, and pancreatic cancer [55]. The combined impact of autophagy inhibition with chemotherapy amplifies the overall anticancer efficacy, resulting in enhanced treatment outcomes. Polymeric micelles, derived from amphiphilic block copolymers, have demonstrated efficacy in targeting the PI3K/AKT/mTOR pathway and regulating autophagy [56]. These nanoparticles encapsulate hydrophobic pharmaceuticals, such as 5-fluorouracil (5-FU) and target their delivery to tumor cells preferentially. Polymeric micelles enhance the therapeutic efficacy of chemotherapy by suppressing autophagy through the mTOR pathway, hence increasing the sensitivity of cancer cells [56]. The combination of autophagy regulation and chemotherapy utilizing polymeric micelles can markedly suppress tumor development in colorectal and gastric malignancies.

Mesoporous silica nanoparticles (MSNs) have arisen as a potent nanocarrier owing to their extensive surface area, adjustable pore dimensions, and capacity to encapsulate both hydrophilic and hydrophobic pharmaceuticals [57]. MSNs influence the PI3K/AKT/mTOR pathway to regulate autophagy, limiting this process through mTOR suppression. The use of chemotherapeutic drugs, such as docetaxel, in conjunction with autophagy inhibition, has demonstrated encouraging outcomes in the management of prostate and lung malignancies. MSNs provide an effective therapeutic method by boosting medication bioavailability and promoting apoptosis via autophagy regulation. Chitosan nanoparticles are synthesized from chitosan, a natural polymer characterized by superior biocompatibility and biodegradability. These nanoparticles can regulate autophagy via the AKT/mTOR signaling pathway. Chitosan nanoparticles, when used in conjunction with doxorubicin, augment the cytotoxicity of chemotherapy by suppressing autophagy, a mechanism frequently employed by cancer cells for protection [58]. This synergistic method has proven beneficial in liver and breast cancer models, resulting in substantial tumor reduction due to amplified chemotherapeutic action. Curcumin, a natural polyphenol, possesses recognized anticancer effects; nevertheless, its therapeutic utility is constrained by inadequate bioavailability. Liposome-encapsulated curcumin improves its therapeutic effectiveness by targeting tumor cells directly. The nanoparticles additionally target the PI3K/AKT/mTOR pathway, suppressing autophagy and enhancing the efficacy of chemotherapy drugs, such as gemcitabine [59]. This combination therapy has demonstrated enhanced apoptosis in pancreatic and breast cancer cells, hence increasing treatment results by circumventing autophagy-induced chemoresistance.

Polyethylenimine (PEI) nanoparticles have exceptional efficacy in the delivery of nucleic acids and small compounds to cells. These nanoparticles regulate the PI3K/AKT/mTOR pathway to suppress autophagy, hence enhancing the susceptibility of cancer cells to chemotherapy [49]. PEI nanoparticles demonstrate synergistic benefits in conjunction with methotrexate, diminishing tumor development and spread in models of leukemia and brain tumors. By modulating the autophagy mechanism, these nanoparticles augment the cytotoxic efficacy of chemotherapy, hence enhancing the overall therapeutic response. Graphene oxide nanoparticles have distinctive characteristics, such as a substantial surface area, flexibility, and the capacity to engage with biological molecules. These nanoparticles can obstruct autophagy via the mTOR pathway, and when administered alongside cisplatin, they enhance the drug’s cytotoxicity [60]. This synergistic effect is especially potent in lung and ovarian malignancies, where the nanoparticles promote apoptosis and diminish chemoresistance, resulting in improved therapeutic outcomes. Nanostructured lipid carriers (NLCs) consist of a solid lipid core encased in a liquid lipid shell, offering superior stability for drug delivery. These nanoparticles can regulate autophagy via the AKT/mTOR pathway, augmenting the efficacy of paclitaxel and other chemotherapy drugs [61]. NLCs demonstrate enhanced anticancer activity in breast, colon, and lung malignancies by simultaneously targeting autophagy and tumor cells. This combined therapy diminishes tumor growth and enhances tumor cell apoptosis by circumventing autophagy-induced chemoresistance.

## 4. Nanoparticles and Their Molecular Mechanisms Modulating PI3K/AKT/mTOR-Mediated Autophagy in Cancer

**Figure 4:**
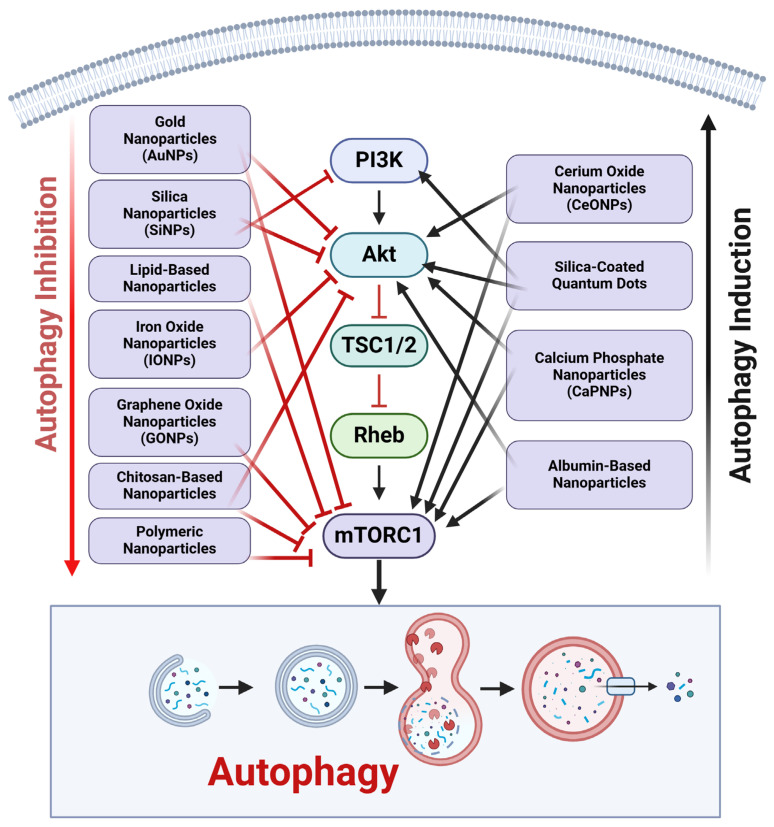
Modulation of PI3K/AKT/mTOR-mediated autophagy by NPs in cancer therapy. The dual function of nanoparticles (NPs) in modulating the PI3K/AKT/mTOR signaling pathway to regulate autophagy in cancer cells. Nanoparticles, including gold nanoparticles (AuNPs), silica nanoparticles (SiNPs), lipid-based nanoparticles, iron oxide nanoparticles (IONPs), graphene oxide nanoparticles (GONPs), chitosan-based nanoparticles, and polymeric nanoparticles, impede the PI3K/AKT/mTOR pathway, resulting in the induction of autophagy. This pathway promotes cancer cell death by elevating autophagic flux, oxidative stress, and apoptosis. Conversely, some nanoparticles, such as cerium oxide nanoparticles (CeONPs), silica-coated quantum dots, calcium phosphate nanoparticles (CaPNPs), and albumin-based nanoparticles stimulate the PI3K/AKT/mTOR pathway, thereby suppressing autophagy. This inhibition diminishes autophagy-related cancer cell survival, hence augmenting the effectiveness of chemotherapeutic drugs. The figure was created using the BioRender.com online commercial platform.

The PI3K/AKT/mTOR signaling pathway is a crucial regulator of autophagy and significantly influences cancer progression. NPs have been developed as a possible therapeutic approach to regulate this system by either promoting or suppressing autophagy in cancer cells (Figure 4).

### 4.1. Nanoparticles That Induce Autophagy via PI3K/AKT/mTOR Inhibition

Gold nanoparticles (AuNPs) impede the phosphorylation of AKT and mTOR, resulting in the induction of autophagy in cancer cells [47]. The inhibition of mTORC1 activity leads to enhanced autophagy flux, facilitating cell death in multiple malignancies, such as breast and lung cancer. Silica nanoparticles (SiNPs) generate oxidative stress and reactive oxygen species (ROS) production, which subsequently downregulates PI3K/AKT signaling [62]. This inhibition stimulates AMPK, suppressing mTOR and facilitating autophagy-induced cancer cell death. Lipid-based nanoparticles encapsulating rapamycin or other mTOR inhibitors directly suppress mTOR activity [63]. These nanoparticles augment autophagy induction, hence increasing the susceptibility of cancer cells to treatment in glioblastoma and ovarian cancer models. Iron oxide nanoparticles (IONPs) promote reactive oxygen species (ROS) production and inhibit the PI3K/AKT/mTOR signaling pathway [64]. This initiates autophagy, resulting in the lysosomal destruction of damaged organelles and proteins, hence augmenting the anticancer efficacy of chemotherapeutics. Graphene oxide nanoparticles (GONPs) inhibit mTORC1 signaling and promote autophagy via disrupting AKT phosphorylation [65]. This method enhances the susceptibility of cancer cells to apoptosis and diminishes tumor proliferation. Chitosan nanoparticles containing bioactive substances, such as curcumin suppress PI3K/AKT signaling [66]. This inhibition diminishes mTOR activity, resulting in increased autophagy flux and apoptotic cell death in hepatocellular cancer. Poly(lactic-co-glycolic acid) (PLGA) nanoparticle-encapsulating medications, like doxorubicin, block the AKT/mTOR pathway, hence inducing autophagy and increasing cancer cell susceptibility to treatment [67].

### 4.2. Nanoparticles That Inhibit Autophagy via PI3K/AKT/mTOR Activation

Cerium oxide nanoparticles (CeONPs) facilitate the activation of AKT and mTOR pathways, thereby suppressing autophagy [68]. This system protects normal cells from harm and diminishes the autophagy-driven survival of cancer cells. Silica-coated quantum dot nanoparticles stimulate the PI3K/AKT/mTOR pathway, inhibiting autophagy in resistant cancer cells [69]. This inhibition reinstates the efficacy of chemotherapeutic drugs in malignancies reliant on autophagy for survival. Calcium phosphate nanoparticles (CaPNPs) stimulate PI3K/AKT signaling and diminish autophagy by augmenting mTOR activity [70]. This inhibition results in the sensitivity of cancer cells to apoptosis generated by chemotherapy. Albumin-based nanoparticles coated with paclitaxel stimulate AKT/mTOR signaling to suppress protective autophagy, hence enhancing apoptosis in pancreatic and breast cancer cells [71].

## 5. Perspectives, Challenges, and Future Directions in Targeting PI3K/AKT/mTOR-Mediated Autophagy Utilizing Nanoparticles

### 5.1. Perspectives

The application of nanotechnology into cancer treatment has transformed targeted medication delivery, especially in regulating autophagy through the PI3K/AKT/mTOR pathway. Nanoparticles (NPs) boost bioavailability, improve pharmacokinetics, and enable controlled drug release, optimizing therapeutic efficacy and reducing systemic toxicity. Their capacity to encapsulate various therapeutic drugs, such as mTOR inhibitors and autophagy modulators, presents intriguing strategies for surmounting resistance mechanisms in cancer cells. The integration of ligand-functionalized nanoparticles facilitates targeted tumor delivery, minimizing off-target effects and increasing drug accumulation in tumor tissues via the increased permeability and retention (EPR) effect [72]. Nonetheless, numerous barriers persist in the translation of nanoparticle-based medicines into clinical practice, and comprehending these impediments is essential for the progression of the area.

### 5.2. Challenges

Despite their potential, several challenges hinder the clinical translation of nanoparticle-based autophagy modulation strategies.

#### 5.2.1. Biocompatibility and Possible Toxicity

A fundamental challenge regarding the utilization of nanoparticles in therapeutic applications is their biocompatibility. While nanoparticles can efficiently transport medications to specific cells, their aggregation in non-target tissues or organs may result in hazardous repercussions [73]. This is particularly crucial when focusing on intracellular networks, such as PI3K/AKT/mTOR, where the exact regulation of autophagy can either facilitate cancer cell apoptosis or unintentionally support tumor viability [18]. Consequently, guaranteeing that nanoparticles are biocompatible, non-toxic, and safely disintegrate within the body constitutes a critical problem to address.

#### 5.2.2. Immune System Recognition and Clearance

Nanoparticles are frequently identified by the immune system as exogenous entities, perhaps leading to their swift elimination prior to arrival at the tumor location. This constrains their therapeutic effectiveness and may result in inadequate sustained medication release at the target site. The immune system’s sensitivity to nanoparticles may provoke inflammatory responses, complicating their application in patients with weakened immune systems [74]. Techniques to alter the surface properties of nanoparticles, including the application of biocompatible polymers or antibodies, can diminish immune recognition and improve their stability in the circulatory system.

#### 5.2.3. Enhancement and Consistency of Nanoparticle Fabrication

The synthesis of nanoparticles for research has advanced significantly, although scaling up production for therapeutic uses is a considerable hurdle. The reproducibility of nanoparticle synthesis is essential for maintaining consistent quality and drug-loading efficacy [75]. Fluctuations in nanoparticle dimensions, surface charge, and composition can profoundly influence their pharmacokinetics, biodistribution, and therapeutic efficacy. Standardizing synthesis techniques and creating economic, large-scale production processes are crucial for the widespread adoption of nanoparticle-based therapeutics in clinical environments.

#### 5.2.4. Tumor Heterogeneity

Tumor heterogeneity poses a distinct obstacle in the advancement of nanoparticle-based therapeutics. Various tumor subtypes or distinct locations within the same tumor may exhibit divergent responses to the inhibition of the PI3K/AKT/mTOR pathway [76]. Moreover, off-target effects, including inadvertent drug release in healthy tissues, may result in toxicities that restrict therapeutic efficacy. Customizing nanoparticle-based therapeutics to address the unique biological attributes of cancers will be crucial in surmounting this barrier.

### 5.3. Future Directions

To address these challenges, future research should focus on the following topics.

#### 5.3.1. Creation of Stimuli-Responsive Nanoparticles for Spatiotemporal Regulation of Drug Release

Subsequent study ought to concentrate on creating nanoparticles capable of responding to stimuli inside the tumor microenvironment. Stimuli-responsive nanoparticles may discharge their therapeutic payload in reaction to variations in pH, temperature, or enzymatic activity, which are frequently modified in neoplastic cells [77]. This method may provide accurate spatiotemporal regulation of drug administration, ensuring that PI3K/AKT/mTOR inhibitors are administered exclusively when and where they are most required, hence reducing toxicity to healthy tissues. Additionally, PI3K/AKT signaling activates HSF-1 (heat shock factor 1), leading to increased expression of HSP70 (heat shock protein 70), which plays a protective role in cancer cells by inhibiting apoptosis and enhancing stress resistance [78]. This mechanism can reduce the effectiveness of photothermal and photodynamic therapies by promoting cell survival under heat or oxidative stress conditions.

#### 5.3.2. Incorporation of Artificial Intelligence and Machine Learning in Nanoparticle Design

The design of nanoparticles for targeted drug administration is a complex process that requires consideration of aspects, such as particle size, surface charge, and drug release characteristics. The incorporation of artificial intelligence (AI) and machine learning (ML) in nanoparticle design could significantly improve the accuracy and efficacy of creating optimum nanoparticles for targeting the PI3K/AKT/mTOR pathway [79]. AI/ML algorithms can be utilized to forecast nanoparticle behavior in biological systems, model their interactions with certain tumor cell types, and refine their design to enhance therapeutic efficacy.

#### 5.3.3. Clinical Trials to Assess Safety and Efficacy in Varied Patient Populations

Although nanoparticle-based medicines exhibit significant potential in preclinical models, their safety and efficacy in human populations require thorough assessment [80]. Future clinical trials ought to concentrate on heterogeneous patient populations to evaluate the potential advantages of targeting PI3K/AKT/mTOR-mediated autophagy across different cancer types. These trials will be essential in assessing the long-term safety of nanoparticles, their capacity to target specific cancers, and their overall therapeutic effectiveness in conjunction with other cancer treatments.

#### 5.3.4. Investigation of Nanoparticles for Customized Cancer Therapy

The primary objective of nanoparticle-based therapeutics is to provide individualized therapy alternatives that are specifically designed to align with the distinct molecular characteristics of each patient’s tumor. By integrating biomarkers associated with PI3K/AKT/mTOR activation, nanoparticles can be engineered to administer tailored medicines to patients most likely to derive benefit [81]. This tailored strategy could markedly enhance treatment results and reduce the negative effects linked to conventional chemotherapy or radiation therapy.

## 6. Conclusions

Nanoparticle-based drugs delivery methods possess significant potential to influence the PI3K/AKT/mTOR pathway and control autophagy in cancer [63]. Progress in nanotechnology and enhanced comprehension of autophagy mechanisms enable the development of more efficacious and individualized cancer treatments. Overcoming current barriers and enhancing nanoparticle design is going to be essential for converting these promising strategies into a possible successful strategy. Therefore, nanoparticle-based treatments possess considerable promise for targeting PI3K/AKT/mTOR-mediated autophagy in cancer therapy. Addressing the challenges of biocompatibility, immune recognition, scalability, and tumor heterogeneity, while investigating future avenues, like stimuli-responsive nanoparticles, AI-driven designs, and personalized medicine, is crucial for actualizing the clinical potential of this novel therapeutic strategy.

## Figures and Tables

**Table 1:** Diverse nanoparticle systems demonstrate the potential of targeted drug delivery to modulate autophagy and enhance the efficacy of cancer therapies.

Nanoparticle Type	Encapsulated/ Incorporated Drug	Target Pathway	Cancer Type	Mechanism	Outcome	Ref.
**Liposomes**	LY294002	PI3K	Breast and prostate cancer	Inhibits PI3K signaling to suppress autophagy and tumor progression.	Enhanced apoptosis and reduced tumor growth.	[33]
**Polymeric nanoparticles**	Rapamycin	mTOR	Glioblastoma	Inhibits mTORC1 to reduce autophagy flux and tumor survival.	Decreased cell proliferation and angiogenesis.	[34]
**Gold nanoparticles**	AKT Inhibitor VIII	AKT	Ovarian cancer	Selectively inhibits AKT phosphorylation to block downstream signaling.	Promotes chemosensitization and autophagic flux suppression.	[35]
**Silica nanoparticles**	LY294002	PI3K	Lung cancer	PI3K inhibition, blocking autophagy, and enhancing chemotherapeutic drug response.	Improved drug delivery and increased cancer cell death.	[36]
**Solid lipid nanoparticles (SLNs)**	Everolimus	mTOR	Pancreatic cancer	Targets mTORC1 to suppress autophagy and tumor cell survival mechanisms.	Enhanced drug bioavailability and tumor suppression.	[37]
**Dendrimers**	Wortmannin	PI3K	Breast cancer	Inhibits autophagy by targeting the PI3K pathway.	Augmented anti-tumor activity and apoptosis.	[38]
**Iron oxide nanoparticles**	Rapamycin	mTOR	Hepatocellular carcinoma	Downregulates mTOR to impair autophagic flux and enhance therapeutic response.	Synergistic reduction in tumor size.	[39]
**PLGA nanoparticles**	MK2206	AKT	Non-small cell lung cancer	AKT inhibition leads to autophagic pathway disruption.	Improved therapeutic efficacy with reduced drug toxicity.	[40]
**Chitosan nanoparticles**	GDC-0941	PI3K	Triple-negative breast cancer	Blocks PI3K-mediated autophagy, enhancing sensitivity to chemotherapy.	Enhanced tumor shrinkage and survival rates.	[41]
**Carbon nanotubes**	Torin 1	mTOR	Colorectal cancer	Dual inhibition of mTORC1 and mTORC2 suppresses autophagy.	Reduced tumor cell viability and improved therapeutic outcomes.	[42]

**Table 2:** Numerous nanoparticles and their siRNA delivery potential for gene therapy, as well as their function in cancer treatment targeting the PI3K/AKT/mTOR-mediated autophagy system.

Nanoparticle Type	Composition	Target	Mechanism of Action	Therapeutic Application	Advantages	Limitations	Ref
**Liposomes**	Phospholipids, cholesterol	PI3K/AKT/mTOR pathway	Encapsulation of siRNA, delivery to cancer cells	Gene silencing of pathway components	Biocompatible, effective gene delivery	Limited stability in circulation	[45]
**Polymeric nanoparticles**	Polyethylene glycol (PEG), PLGA	PI3K, AKT, mTOR	Controlled release of siRNA to inhibit PI3K/AKT/mTOR	Inhibition of autophagy-related genes	Long circulation time, high drug loading	Potential toxicity of polymer components	[46]
**Gold nanoparticles**	Gold core with functionalized shell	mTOR, AKT	Delivery of siRNA for gene silencing	Targeted silencing of mTOR/AKT pathway	Easy functionalization, enhanced stability	Potential for cytotoxicity at high doses	[47]
**Dendrimers**	Branched polymers, PEG	PI3K, AKT, mTOR	Efficient siRNA loading, targeting tumor cells	Gene therapy for PI3K/AKT pathway in cancers	Well-defined structure, high loading capacity	Complex synthesis, limited biodegradability	[48]
**Mesoporous silica nanoparticles (MSNs)**	Silica, functionalized surface	PI3K, AKT, mTOR	Encapsulation of siRNA, controlled release	Combination therapy with gene silencing	High surface area, tunable pore size	Potential for inflammation due to silica material	[49]
**Polymeric micelles**	Amphiphilic copolymers	PI3K/AKT/mTOR	siRNA encapsulation in micellar core, controlled release	Targeted delivery of gene silencing agents	Low toxicity, good pharmacokinetics	Limited stability in blood circulation	[50]
**Solid lipid nanoparticles (SLNs)**	Solid lipid core, surfactants	PI3K, AKT	siRNA loading in lipid matrix, targeting cancer cells	RNAi therapy for PI3K/AKT/mTOR pathway modulation	High stability, biocompatibility	Limited drug-loading capacity	[51]
**Calcium phosphate nanoparticles**	Calcium phosphate, PEG	AKT, mTOR	Encapsulation of siRNA, gene delivery via calcium phosphate	Gene silencing of PI3K/AKT in cancer	Biodegradable, efficient encapsulation	Potential for low release rates	[36]
**Graphene oxide nanoparticles**	Graphene oxide, functionalized surface	PI3K, AKT, mTOR	Loading of siRNA on surface, gene silencing effect	Targeted cancer therapy through pathway inhibition	High surface area, biocompatibility	Cytotoxicity at high doses, aggregation risk	[52]
**Nanogels**	Hydrogels with crosslinked polymers	mTOR, PI3K, AKT	Encapsulation of siRNA in nanogel, targeted delivery	Modulation of autophagy-related signaling in cancer	High biocompatibility, tunable release rates	Limited cellular uptake without surface modification	[53]

**Table 3:** Demonstrates the potential of integrating autophagy modulation by nanoparticle-based drug delivery systems with chemotherapeutic drugs to improve the efficacy of cancer treatment.

Nanoparticle	Targeted Pathway	Autophagy Modulation	Chemotherapeutic Agent	Synergistic Effect	Cancer Type	Ref
**Doxorubicin-loaded liposomes**	PI3K/AKT/mTOR	Inhibits autophagy via the mTOR pathway	Doxorubicin	Enhanced cell death by reducing chemoresistance and promoting apoptosis	Breast, lung, ovarian	[54]
**Polymeric nanoparticles (PNPs)**	PI3K/AKT/mTOR	Autophagy inhibition by mTOR suppression	Paclitaxel	Increased therapeutic efficacy by synergizing autophagy inhibition and chemotherapy	Colon, lung, prostate	[35]
**Gold nanoparticles (AuNPs)**	AKT/mTOR	mTOR inhibition	Cisplatin	Enhanced cytotoxicity via decreased autophagy and enhanced chemotherapy response	Lung, ovarian, pancreatic	[55]
**Polymeric micelles**	PI3K/AKT/mTOR	Modulates autophagy via the AKT/mTOR axis	5-FU (fluorouracil)	Synergistic effects on tumor growth inhibition by blocking autophagy	Colorectal, gastric	[56]
**Mesoporous silica nanoparticles (MSNs)**	PI3K/AKT/mTOR	mTOR-dependent autophagy suppression	Docetaxel	Enhanced therapeutic response through autophagy inhibition and drug delivery	Prostate, lung	[57]
**Chitosan nanoparticles**	PI3K/AKT/mTOR	AKT-mediated autophagy modulation	Doxorubicin	Synergistic cytotoxic effects by preventing autophagy-induced survival	Liver, breast	[58]
**Liposome-encapsulated curcumin**	PI3K/AKT/mTOR	Inhibits autophagy by mTOR activation	Gemcitabine	Enhanced apoptosis by modulating autophagy and chemotherapeutic response	Pancreatic, breast	[59]
**Polyethylenimine (PEI) nanoparticles**	PI3K/AKT/mTOR	Modulates autophagy via the PI3K/AKT pathway	Methotrexate	Synergistic effect in inhibiting tumor growth and metastasis	Leukemia, brain tumors	[49]
**Graphene oxide nanoparticles**	AKT/mTOR	Autophagy inhibition via the mTOR pathway	Cisplatin	Enhanced tumor cell apoptosis through autophagy modulation and drug synergy	Lung, ovary	[60]
**Nanostructured lipid carriers (NLCs)**	PI3K/AKT/mTOR	Inhibits autophagy through AKT/mTOR signaling	Paclitaxel	Enhanced anticancer effect by modulating autophagy and drug resistance	Breast, colon, lung	[61]

## References

[1] Chun Y., Kim J. (2018). Autophagy: An essential degradation program for cellular homeostasis and life. Cells.

[2] Rahman M.A., Shaikh M.H., Gupta R.D., Siddika N., Shaikh M.S., Zafar M.S., Kim B., Hoque Apu E. (2024). Advancements in Autophagy Modulation for the Management of Oral Disease: A Focus on Drug Targets and Therapeutics. Biomedicines.

[3] Yun C.W., Jeon J., Go G., Lee J.H., Lee S.H. (2020). The dual role of autophagy in cancer development and a therapeutic strategy for cancer by targeting autophagy. Int. J. Mol. Sci..

[4] Morgos D.-T., Stefani C., Miricescu D., Greabu M., Stanciu S., Nica S., Stanescu-Spinu I.-I., Balan D.G., Balcangiu-Stroescu A.-E., Coculescu E.-C. (2024). Targeting PI3K/AKT/mTOR and MAPK signaling pathways in gastric cancer. Int. J. Mol. Sci..

[5] Xu Z., Han X., Ou D., Liu T., Li Z., Jiang G., Liu J., Zhang J. (2020). Targeting PI3K/AKT/mTOR-mediated autophagy for tumor therapy. Appl. Microbiol. Biotechnol..

[6] Piekarski A.L. (2015). Autophagy and its Potential Role in Stress and Feed Efficiency Using Avian Lines.

[7] Jorissen R.N., Walker F., Pouliot N., Garrett T.P., Ward C.W., Burgess A.W. (2003). Epidermal growth factor receptor: Mechanisms of activation and signalling. EGF Recept. Fam..

[8] Ji S., Lin W., Wang L., Ni Z., Jin F., Zha X., Fei G. (2017). Combined targeting of mTOR and Akt using rapamycin and MK-2206 in the treatment of tuberous sclerosis complex. J. Cancer.

[9] Marzoog B.A. (2022). Autophagy in cancer cell transformation: A potential novel therapeutic strategy. Curr. Cancer Drug Targets.

[10] Miricescu D., Totan A., Stanescu-Spinu I.-I., Badoiu S.C., Stefani C., Greabu M. (2020). PI3K/AKT/mTOR signaling pathway in breast cancer: From molecular landscape to clinical aspects. Int. J. Mol. Sci..

[11] Chen Y., Wei H., Liu F., Guan J.-L. (2014). Hyperactivation of mammalian target of rapamycin complex 1 (mTORC1) promotes breast cancer progression through enhancing glucose starvation-induced autophagy and Akt signaling. J. Biol. Chem..

[12] Shi F., Luo D., Zhou X., Sun Q., Shen P., Wang S. (2021). Combined effects of hyperthermia and chemotherapy on the regulate autophagy of oral squamous cell carcinoma cells under a hypoxic microenvironment. Cell Death Discov..

[13] Li H., Wen X., Ren Y., Fan Z., Zhang J., He G., Fu L. (2024). Targeting PI3K family with small-molecule inhibitors in cancer therapy: Current clinical status and future directions. Mol. Cancer.

[14] Tufail M., Wan W.-D., Jiang C., Li N. (2024). Targeting PI3K/AKT/mTOR Signaling to Overcome Drug Resistance in Cancer. Chem. Biol. Interact..

[15] Zhou H., Huang S. (2010). The complexes of mammalian target of rapamycin. Curr. Protein Pept. Sci..

[16] Rahman M.A., Cho Y., Nam G., Rhim H. (2021). Antioxidant compound, oxyresveratrol, inhibits APP production through the AMPK/ULK1/mTOR-mediated autophagy pathway in mouse cortical astrocytes. Antioxidants.

[17] Li D., Geng D., Wang M. (2024). Advances in natural products modulating autophagy influenced by cellular stress conditions and their anticancer roles in the treatment of ovarian cancer. FASEB J..

[18] Glaviano A., Foo A.S., Lam H.Y., Yap K.C., Jacot W., Jones R.H., Eng H., Nair M.G., Makvandi P., Geoerger B. (2023). PI3K/AKT/mTOR signaling transduction pathway and targeted therapies in cancer. Mol. Cancer.

[19] Elumalai K., Srinivasan S., Shanmugam A. (2024). Review of the efficacy of nanoparticle-based drug delivery systems for cancer treatment. Biomed. Technol..

[20] Choi J., Kim B.H. (2024). Ligands of Nanoparticles and Their Influence on the Morphologies of Nanoparticle-Based Films. Nanomaterials.

[21] Sajeevan D., Are R.P., Hota P., Babu A.R. (2025). Nanoparticles as Drug Delivery Carrier-synthesis, Functionalization and Application. Curr. Pharm. Des..

[22] Hristova-Panusheva K., Xenodochidis C., Georgieva M., Krasteva N. (2024). Nanoparticle-Mediated Drug Delivery Systems for Precision Targeting in Oncology. Pharmaceuticals.

[23] Geng Y., Zou H., Li Z., Wu H. (2024). Recent advances in nanomaterial-driven strategies for diagnosis and therapy of vascular anomalies. J. Nanobiotechnol..

[24] Dehchani A.J., Jafari A., Shahi F. (2024). Nanogels in Biomedical Engineering: Revolutionizing Drug Delivery, Tissue Engineering, and Bioimaging. Polym. Adv. Technol..

[25] Alharbi H.M., Alqahtani T., Alamri A.H., Kumarasamy V., Subramaniyan V., Babu K.S. (2024). Nanotechnological synergy of mangiferin and curcumin in modulating PI3K/Akt/mTOR pathway: A novel front in ovarian cancer precision therapeutics. Front. Pharmacol..

[26] Jirandehi A.K., Asgari R., Shahbaz S.K., Rezaei N. (2024). Nanomedicine marvels: Crafting the future of cancer therapy with innovative statin nano-formulation strategies. Nanoscale Adv..

[27] Sunoqrot S., Abusulieh S., Sabbah D. (2024). Polymeric Nanoparticles Potentiate the Anticancer Activity of Novel PI3Kα Inhibitors Against Triple-Negative Breast Cancer Cells. Biomedicines.

[28] Paul G., Gupta U., Shah H., Mazahir F., Yadav A. (2024). Inorganic and metal-based nanoparticles. Molecular Pharmaceutics and Nano Drug Delivery.

[29] Oehler J.B., Rajapaksha W., Albrecht H. (2024). Emerging applications of nanoparticles in the diagnosis and treatment of breast cancer. J. Pers. Med..

[30] Debnath S.K., Debnath M., Ghosh A., Srivastava R., Omri A. (2024). Targeting Tumor Hypoxia with Nanoparticle-Based Therapies: Challenges, Opportunities, and Clinical Implications. Pharmaceuticals.

[31] Darwish M.A., Abd-Elaziem W., Elsheikh A., Zayed A.A. (2024). Advancements in Nanomaterials for Nanosensors: A Comprehensive Review. Nanoscale Adv..

[32] Mirabdali S., Ghafouri K., Farahmand Y., Gholizadeh N., Yazdani O., Esbati R., Hajiagha B.S., Rahimi A. (2024). The role and function of autophagy through signaling and pathogenetic pathways and lncRNAs in ovarian cancer. Pathol. Res. Pract..

[33] Tang M., Zhang Z., Wang P., Zhao F., Miao L., Wang Y., Li Y., Li Y., Gao Z. (2024). Advancements in precision nanomedicine design targeting the anoikis-platelet interface of circulating tumor cells. Acta Pharm. Sin. B.

[34] Jia L., Hao S.-L., Yang W.-X. (2020). Nanoparticles induce autophagy via mTOR pathway inhibition and reactive oxygen species generation. Nanomedicine.

[35] Liu Z., Lu T., Qian R., Wang Z., Qi R., Zhang Z. (2024). Exploiting Nanotechnology for Drug Delivery: Advancing the Anti-Cancer Effects of Autophagy-Modulating Compounds in Traditional Chinese Medicine. Int. J. Nanomed..

[36] Tavakol S., Ashrafizadeh M., Deng S., Azarian M., Abdoli A., Motavaf M., Poormoghadam D., Khanbabaei H., Ghasemipour Afshar E., Mandegary A. (2019). Autophagy modulators: Mechanistic aspects and drug delivery systems. Biomolecules.

[37] Kargozar S., Baino F., Hamzehlou S., Hamblin M.R., Mozafari M. (2020). Nanotechnology for angiogenesis: Opportunities and challenges. Chem. Soc. Rev..

[38] Negi S., Chaudhuri A., Kumar D.N., Dehari D., Singh S., Agrawal A.K. (2022). Nanotherapeutics in autophagy: A paradigm shift in cancer treatment. Drug Deliv. Transl. Res..

[39] Badawy M.M., Abdel-Hamid G.R., Mohamed H.E. (2023). Antitumor activity of chitosan-coated iron oxide nanocomposite against hepatocellular carcinoma in animal models. Biol. Trace Elem. Res..

[40] Mendes R., Carreira B., Baptista P.V., Fernandes A.R. (2016). Non-small cell lung cancer biomarkers and targeted therapy-two faces of the same coin fostered by nanotechnology. Expert Rev. Precis. Med. Drug Dev..

[41] Sharma V., Sharma A.K., Punj V., Priya P. (2019). Recent nanotechnological interventions targeting PI3K/Akt/mTOR pathway: A focus on breast cancer. Seminars in Cancer Biology.

[42] Ihlamur M., Akgül B., Zengin Y., Korkut Ş.V., Kelleci K., Abamor E.Ş. (2024). The mTOR Signaling pathway and mTOR Inhibitors in cancer: Next-generation inhibitors and approaches. Curr. Mol. Med..

[43] Yang Y., Liu L., Tian Y., Gu M., Wang Y., Ashrafizadeh M., Aref A.R., Cañadas I., Klionsky D.J., Goel A. (2024). Autophagy-driven regulation of cisplatin response in human cancers: Exploring molecular and cell death dynamics. Cancer Lett..

[44] Mahajan S., Aalhate M., Guru S.K., Singh P.K. (2022). Nanomedicine as a magic bullet for combating lymphoma. J. Control. Release.

[45] Walweel N., Aydin O. (2024). Enhancing therapeutic efficacy in cancer treatment: Integrating nanomedicine with autophagy inhibition strategies. ACS Omega.

[46] Paskeh M.D.A., Entezari M., Clark C., Zabolian A., Ranjbar E., Farahani M.V., Saleki H., Sharifzadeh S.O., Far F.B., Ashrafizadeh M. (2022). Targeted regulation of autophagy using nanoparticles: New insight into cancer therapy. Biochim. Biophys. Acta Mol. Basis Dis..

[47] Elmetwalli A., El-Sewedy T., Hassan M.G., Abdel-Monem M.O., Hassan J., Ismail N.F., Salama A.F., Fu J., Mousa N., Sabir D.K. (2024). Gold nanoparticles mediate suppression of angiogenesis and breast cancer growth via MMP-9/NF-κB/mTOR and PD-L1/PD-1 signaling: Integrative in vitro validation and network pharmacology insights. Naunyn-Schmiedeberg’s Arch. Pharmacol..

[48] Dong Y., Yu T., Ding L., Laurini E., Huang Y., Zhang M., Weng Y., Lin S., Chen P., Marson D. (2018). A dual targeting dendrimer-mediated siRNA delivery system for effective gene silencing in cancer therapy. J. Am. Chem. Soc..

[49] Zhang L., Feng G., Yang S., Liu B., Niu Y., Fan P., Liu Z., Chen J., Cui L., Zhou G. (2021). Polyethylenimine-modified mesoporous silica nanoparticles induce a survival mechanism in vascular endothelial cells via microvesicle-mediated autophagosome release. ACS Nano.

[50] Godakhindi V., Tarannum M., Dam S.K., Vivero-Escoto J.L. (2024). Mesoporous Silica Nanoparticles as an Ideal Platform for Cancer Immunotherapy: Recent Advances and Future Directions. Adv. Healthc. Mater..

[51] Scioli Montoto S., Muraca G., Ruiz M.E. (2020). Solid Lipid Nanoparticles for Drug Delivery: Pharmacological and Biopharmaceutical Aspects. Front. Mol. Biosci..

[52] Salama A.M., Yasin G., Zourob M., Lu J. (2022). Fluorescent Biosensors for the Detection of Viruses Using Graphene and Two-Dimensional Carbon Nanomaterials. Biosensors.

[53] Neerooa B., Ooi L.T., Shameli K., Dahlan N.A., Islam J.M.M., Pushpamalar J., Teow S.Y. (2021). Development of Polymer-Assisted Nanoparticles and Nanogels for Cancer Therapy: An Update. Gels.

[54] Rawat P.S., Jaiswal A., Khurana A., Bhatti J.S., Navik U. (2021). Doxorubicin-induced cardiotoxicity: An update on the molecular mechanism and novel therapeutic strategies for effective management. Biomed. Pharmacother..

[55] Ning L., Zhu B., Gao T. (2017). Gold Nanoparticles: Promising Agent to Improve the Diagnosis and Therapy of Cancer. Curr. Drug Metab..

[56] Sanaei M.J., Baghery Saghchy Khorasani A., Pourbagheri-Sigaroodi A., Shahrokh S., Zali M.R., Bashash D. (2022). The PI3K/Akt/mTOR axis in colorectal cancer: Oncogenic alterations, non-coding RNAs, therapeutic opportunities, and the emerging role of nanoparticles. J. Cell Physiol..

[57] Narayan R., Nayak U.Y., Raichur A.M., Garg S. (2018). Mesoporous Silica Nanoparticles: A Comprehensive Review on Synthesis and Recent Advances. Pharmaceutics.

[58] Sharifi-Rad J., Quispe C., Butnariu M., Rotariu L.S., Sytar O., Sestito S., Rapposelli S., Akram M., Iqbal M., Krishna A. (2021). Chitosan nanoparticles as a promising tool in nanomedicine with particular emphasis on oncological treatment. Cancer Cell Int..

[59] Zahedipour F., Bolourinezhad M., Teng Y., Sahebkar A. (2021). The Multifaceted Therapeutic Mechanisms of Curcumin in Osteosarcoma: State-of-the-Art. J. Oncol..

[60] Thakur S., Bi A., Mahmood S., Samriti, Ruzimuradov O., Gupta R., Cho J., Prakash J. (2024). Graphene oxide as an emerging sole adsorbent and photocatalyst: Chemistry of synthesis and tailoring properties for removal of emerging contaminants. Chemosphere.

[61] Chauhan I., Yasir M., Verma M., Singh A.P. (2020). Nanostructured Lipid Carriers: A Groundbreaking Approach for Transdermal Drug Delivery. Adv. Pharm. Bull..

[62] Liu W., Liu H., Zhang S., Hao H., Meng F., Ma W., Guo Z., Jiang S., Shang X. (2024). Silica nanoparticles cause ovarian dysfunction and fertility decrease in mice via oxidative stress-activated autophagy and apoptosis. Ecotoxicol. Environ. Saf..

[63] Yoon M.S. (2020). Nanotechnology-Based Targeting of mTOR Signaling in Cancer. Int. J. Nanomed..

[64] Yu S., Tong L., Shen J., Li C., Hu Y., Feng K., Shao J. (2024). Recent research progress based on ferroptosis-related signaling pathways and the tumor microenvironment on it effects. Eur. J. Med. Chem..

[65] Wang W., Su Y., Qi R., Li H., Jiang H., Li F., Li B., Sun H. (2024). Indoxacarb triggers autophagy and apoptosis through ROS accumulation mediated by oxidative phosphorylation in the midgut of Bombyx mori. Pestic. Biochem. Physiol..

[66] Mishra B., Yadav A.S., Malhotra D., Mitra T., Sinsinwar S., Radharani N.N.V., Sahoo S.R., Patnaik S., Kundu G.C. (2024). Chitosan Nanoparticle-Mediated Delivery of Curcumin Suppresses Tumor Growth in Breast Cancer. Nanomaterials.

[67] Wang X., Wang L., Hao Q., Cai M., Wang X., An W. (2024). Harnessing glucose metabolism with nanomedicine for cancer treatment. Theranostics.

[68] Sun X., Xu X., Yue X., Wang T., Wang Z., Zhang C., Wang J. (2024). Nanozymes with Osteochondral Regenerative Effects: An Overview of Mechanisms and Recent Applications. Adv. Healthc. Mater..

[69] Fan Z., Shao Y., Jiang X., Zhou J., Yang L., Chen H., Liu W. (2024). Cytotoxic effects of NIR responsive chitosan-polymersome layer coated melatonin-upconversion nanoparticles on HGC27 and AGS gastric cancer cells: Role of the ROS/PI3K/Akt/mTOR signaling pathway. Int. J. Biol. Macromol..

[70] Rafieerad A., Saleth L.R., Khanahmadi S., Amiri A., Alagarsamy K.N., Dhingra S. (2025). Periodic Table of Immunomodulatory Elements and Derived Two-Dimensional Biomaterials. Adv. Sci..

[71] Yang C., Ding Y., Mao Z., Wang W. (2024). Nanoplatform-Mediated Autophagy Regulation and Combined Anti-Tumor Therapy for Resistant Tumors. Int. J. Nanomed..

[72] Zhang J., Wang S., Zhang D., He X., Wang X., Han H., Qin Y. (2023). Nanoparticle-based drug delivery systems to enhance cancer immunotherapy in solid tumors. Front. Immunol..

[73] Joseph T.M., Kar Mahapatra D., Esmaeili A., Piszczyk Ł., Hasanin M.S., Kattali M., Haponiuk J., Thomas S. (2023). Nanoparticles: Taking a Unique Position in Medicine. Nanomaterials.

[74] Liu J., Liu Z., Pang Y., Zhou H. (2022). The interaction between nanoparticles and immune system: Application in the treatment of inflammatory diseases. J. Nanobiotechnol..

[75] Piscatelli J.A., Ban J., Lucas A.T., Zamboni W.C. (2021). Complex Factors and Challenges that Affect the Pharmacology, Safety and Efficacy of Nanocarrier Drug Delivery Systems. Pharmaceutics.

[76] Liu S., Liu Z., Lei H., Miao Y.B., Chen J. (2024). Programmable Nanomodulators for Precision Therapy, Engineering Tumor Metabolism to Enhance Therapeutic Efficacy. Adv. Healthc. Mater..

[77] Pham S.H., Choi Y., Choi J. (2020). Stimuli-Responsive Nanomaterials for Application in Antitumor Therapy and Drug Delivery. Pharmaceutics.

[78] Wong S.H.D., Yin B., Li Z., Yuan W., Zhang Q., Xie X., Tan Y., Wong N., Zhang K., Bian L. (2023). Mechanical manipulation of cancer cell tumorigenicity via heat shock protein signaling. Sci. Adv..

[79] Nag S., Mitra O., Tripathi G., Adur I., Mohanto S., Nama M., Samanta S., Gowda B.H.J., Subramaniyan V., Sundararajan V. (2024). Nanomaterials-assisted photothermal therapy for breast cancer: State-of-the-art advances and future perspectives. Photodiagnosis Photodyn. Ther..

[80] Ragelle H., Danhier F., Préat V., Langer R., Anderson D.G. (2017). Nanoparticle-based drug delivery systems: A commercial and regulatory outlook as the field matures. Expert. Opin. Drug Deliv..

[81] Tufail M., Hu J.J., Liang J., He C.Y., Wan W.D., Huang Y.Q., Jiang C.H., Wu H., Li N. (2024). Predictive, preventive, and personalized medicine in breast cancer: Targeting the PI3K pathway. J. Transl. Med..

